# Effect of the COVID-19 Pandemic on Nonsurgical Cosmetic Procedure Interest

**DOI:** 10.1177/07488068221141168

**Published:** 2022-12-20

**Authors:** Melinda Lem, Jason T. Pham, Joshua KyungHo Kim, Cathy J. Tang

**Affiliations:** 1 University of California Irvine, Orange, USA; 2 Duke University School of Medicine, Durham, NC, USA

**Keywords:** chemical peels, skin filler substances, botox, facial cosmetic surgery, laser resurfacing

## Abstract

The COVID-19 pandemic caused many shifts in the national economy, job market, and healthcare sector, especially elective procedures such as in cosmetic plastic surgery. While there has been research in the changes in cosmetic plastic surgery since the start of the COVID-19 pandemic, there have been no study to date determining the changes in distinct sectors, such as nonsurgical plastic surgery procedures and the popular trends that may be more sensitive to changes to general public interest. The Google Trends tool was used to determine these changes in interest. Nonsurgical procedures were retrieved from the American Society of Plastic Surgery (ASPS) website, grouped into categories, such as neurotoxin, chemical peel, filler, hair removal, dermaplaning, skin laser treatment, skin tightening, and thread lift, and used as Google Trends search terms. Search term weekly data were collected from March 2018 to March 2020 and March 2020 to March 2022, divided by the start of the COVID-19 lockdown, and compared. After the start of the COVID-19 pandemic, the majority of nonsurgical procedures showed an increase in interest, with the most significant changes including neurotoxin procedures especially lip Botox, filler in the chin, cheek, jawline, tear trough, lip, and nose, skin-improving treatments, non-invasive forms of body contouring, and thread lift. Brands like Dysport, Radiesse, Restylane, Sculptra, Versa, Emsculpt, and Morpheus8 showed increased interest. Most nonsurgical procedures increased in interest after the COVID-19 lockdown, with facial and skin-improving procedures showing the most increase in interest.

## Introduction

The World Health Organization declared COVID-19 a pandemic in March 2020, marking an unprecedented time in the medical system.^1,2^ Healthcare services were restricted, with a majority of states publishing recommendations limiting to only essential surgeries.^3^ This led to a steep decrease in the interest and volume of elective and reconstructive plastic surgery procedures.^4-6^ Not only did plastic surgeons cease plastic surgery procedures, but they also ceased nonsurgical procedures such as laser treatments and injectables. However, interest quickly recovered and reached new heights, especially in the post-COVID-19 vaccine period, possibly due to the increase of virtual meetings, increased time in front of a camera focused on facial features, time at home, and social media use.^7-9^

Interest in nonsurgical procedures, particularly elective ones, may have followed the same overall trends. Due to the accessibility of nonsurgical procedures, a lesser commitment, and affordability, patients new to plastic surgery may be more likely to try this instead of trying a more invasive surgical procedure. One way to evaluate public interest trends is through the now commonly used Google Trends Search Tool.^10^

The Google Trends Search Tool is a public database that reports relative interest and volume of online searches from consumers’ Google Search inputs.^10^ It is a powerful resource for understanding the general public’s current interest, knowledge, or awareness about certain terms or topics. Moreover, the Trends database is highly accessible, enabling researchers to quickly access historical search data within a chosen timeframe and geographic region. Multiple studies have utilized Google Trends to investigate the increased interest in plastic surgery and cosmetic procedures in the wake of the COVID-19 pandemic in the United States.^8,11^ Of relevance, Tijerina et al^12,13^ showed that the volume of many nonsurgical plastic surgery procedures was significantly correlated to Google Trends search interest. Since the usefulness of Google Trends has been established in similar papers, this study will analyze Google Trends data to characterize how the general public’s interest in nonsurgical procedures changed following the COVID-19 pandemic.

At this time, no studies have made clear distinctions between surgical or nonsurgical procedures while investigating trends after the implementation of the COVID-19 vaccine. In this study, we seek to address changing interest in nonsurgical plastic surgery procedures following the pandemic. Understanding these trends should help physicians understand which particular procedures, devices, and technologies are worth investing in to satisfy patient interests and needs.

## Materials and Methods

### Data Collection

The most common nonsurgical procedures were retrieved from the American Society of Plastic Surgery (ASPS) website and corroborated with terms used by RealSelf.^14^ The Google Trends Search Tool was used to examine these procedures as individual search terms, which are exact Google searches by the general public. Since the search terms are verbatim, related and similar iterations of the procedure name were also searched using Boolean logic (AND) to ensure that results were not missed. Related terms were grouped into general categories, such as neurotoxin, chemical peel, filler, hair removal, dermaplaning, skin laser treatment, skin tightening, and thread lift. These searches were restricted to “web” results from “all categories” within the United States. Data were captured weekly from March 18, 2018 to March 13, 2022, a 4-year period that encompassed 2 years before and after the United States lockdown from COVID-19 began on March 13, 2020.

Google Trends search results are provided on a scale from 0 to 100, with 100 indexed as the highest search volume for that procedure in a given time period and specified country. Therefore, these results are normalized to only the search term used and not comparable to separate searches. Google Trend data only reveal information about trend over time, not overall volume, prevalence, or comparison data.^15^

### Data Analysis

To compare the means between the before and after COVID-19 peak period, a 2-tailed Mann-Whitney U test was performed to determine significance. Student’s *t*-test and Wilcoxon Signed Rank test were not performed as equal variance and normality could not be guaranteed by Levene’s Test and Shapiro-Wilk test, respectively. Our significance was evaluated at *P* < .05. Effect size of the Mann-Whitney U test was also calculated. Values between 0.3 and 0.5 were moderate, and values exceeding 0.5 were classified as large effects. Analysis was conducted by using SPSS (IBM Corp. Released 2020. IBM SPSS Statistics Version 27.0. Armonk, NY, USA).

## Results

### Overall Trends

The majority of nonsurgical procedures showed a statistically significant increase in interest when comparing before and after the peak of the COVID-19 pandemic. The procedure categories with the most significant changes included neurotoxin procedures, filler, skin care treatments, non-invasive forms of body contouring (i.e. Emsculpt, Coolsculpting), and thread lift. Each subcategory was analyzed independent of each other.

### Procedure Specific Trends

**Figure 1.**
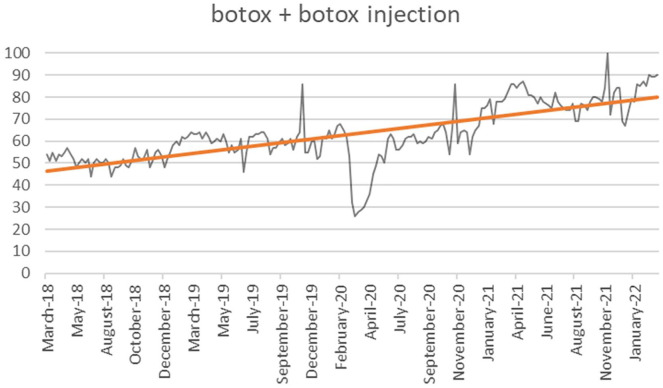
Visual representation of Botox + Botox injection interest trend over time.

Many procedures had increasing interest trends with large magnitudes (Table 1). Neurotoxin procedures were found to be primarily significant with Botox + Botox injection (*P* < .001, *r* = 0.639), “lip flip” + lip Botox (*P* < .001, *r* = 0.894) and Dysport (*P* < .001, *r* = 0.553), with less of a trend from Jeuveau and Xeomin (Figure 1). Chemical peel was only significant as a generalized term, but not when including specific chemicals or brands (*P* < .001, *r* = 0.519). Filler terms were significant when discussing specific locations including the cheek, chin, jawline, lip, tear trough, and nose (*P* < .001, *r* ≥ 0.5). However, marionette lines and nasolabial fillers showed no difference (*P* > .05). Specific filler brands were also significant, including Radiesse, Restylane, Sculptra, and Versa (*P* < .001, *r* ≥ 0.5). Laser hair removal was also significant (*P* < .001, *r* = 0.632). When analyzing dermaplaning, only diamond facial/microdermabrasion was significant in its increase over time (*P* < .001, *r* = 0.596). Nonsurgical body contouring and skin tightening procedures showed that laser liposuction, Emsculpt, and Morpheus8 of radiofrequency lipolysis to be significant (*P* < .001, *r* ≥ 0.5). Kybella was significantly increased with a moderate effect size (*P* < .001, *r* = 0.401). Thread lift, halo laser treatment, and microneedling with radiofrequency or platelet rich plasma were found to be significant as well (*P* < .001, *r* ≥ 0.5).

**Table 1.** Increasing Nonsurgical Procedure Interest Trends When Comparing Before and After the Start of the COVID-19 Pandemic.

Category	Statistic	*P*-value	Effect size	Effect magnitude
Neurotoxin				
Botox^®^ + Botox^®^ injection	1970	< .001	0.6392	High
Dysport^®^	2439	< .001	0.5534	High
Jeuveau^®^ + “newtox”	4311	.004	0.2104	Low
Xeomin^®^	3050	< .001	0.4415	Moderate
“Lip flip” + lip botox	578	< .001	0.8942	High
Chemical peel				
Phenol skin peel + phenol chemical peel + deep chemical peel	4249	.003	0.2219	Low
Chemical peel + chemical skin peel	2625	< .001	0.5193	High
Jessner peel + Jessner chemical peel	5194	.271	0.0488	Low
Filler				
Hyaluronic acid filler	1122	< .001	0.7945	High
Cheek filler	1184	< .001	0.7832	High
Chin filler	716	< .001	0.8690	High
Jawline filler	894	< .001	0.8363	High
Lip filler + lip augmentation	516	< .001	0.9055	High
Under eye filler + tear trough filler	1758	< .001	0.6781	High
“Liquid rhinoplasty” + nonsurgical rhinoplasty + nonsurgical nose job	1070	< .001	0.8040	High
Marionette lines filler + nasolabial filler	2775	< .001	0.4919	Moderate
Radiesse^®^	3711	< .001	0.3204	Moderate
Restylane^®^ + kysse	2510	< .001	0.5403	High
Sculptra^®^	1425	< .001	0.7390	High
Versa^™^	2238	< .001	0.5902	High
Dermal filler	2324	< .001	0.5745	High
Hair removal				
Laser hair removal	2007	< .001	0.6325	High
Alma Soprano ICE^™^	4996	.117	0.0850	Low
Dermaplaning				
Dermaplaning + dermaplane + dermabrasion	4887	.095	0.1050	Low
“Diamond facial” + “diamond microdermabrasion”	2204	< .001	0.5964	High
Laser Lipo				
Laser liposuction + “laser lipo”	2618	< .001	0.5206	High
Emsculpt				
Emsculpt	2458	< .001	0.5498	High
Kybella^®^				
Kybella^®^	3267	< .001	0.4017	Moderate
Radiofrequency Lipolysis/skin tightening				
Radiofrequency skin tightening	3570	< .001	0.3462	Moderate
Evolve body contouring	3232	< .001	0.4081	Moderate
Morpheus8	677	< .001	0.8760	High
Bodytite	3647	< .001	0.3321	Moderate
Accutite	3827	< .001	0.2991	
Facetite	3256	< .001	0.4038	Moderate
Thread lift				
Nonsurgical facelift + nonsurgical eye lift + “thread lift” + thread facelift	617	< .001	0.8870	High
“O thread”	638	< .001	0.8832	High
Skin laser treatment				
Erbium laser resurfacing	5030	.16	0.0788	Low
CO2 laser resurfacing	2907	< .001	0.4676	Moderate
Laser skin treatment + laser skin resurfacing + laser skin tightening + acne scar laser treatment	4969	.131	0.0900	Low
Picosure^®^ + Picofocus^®^	5301	.358	0.0291	Low
Broadband light therapy	5344	.606	0.0213	Low
Halo^®^ laser treatment	2487	< .001	0.5446	High
Cellulite treatment				
Cellulite treatment	3244	< .001	0.4059	Moderate
Microneedling				
Microneedling + microneedling PRP^*^ + microneedling platelet rich plasma	2517	< .001	0.5391	High
Microneedling radiofrequency	2338	< .001	0.5719	High
Microneedling Vivace^™^	5186	.265	0.0503	Low
Aquagold^®^ facial	3854	< .001	0.2941	Low
Tattoo removal				
Tattoo removal	3078	< .001	0.4364	Moderate

* PRP = Platelet-rich plasma

Our analysis identified decreases in search interest for some filler brands like Juvederm and Bellafill (*P* = 1), Coolsculpting (*P* = 1), various skin laser treatments such as fraxel and intense pulsed light (IPL) (*P* = .922, *P* = 1), and Cellfina (*P* = .999). None of these decreasing trends were significant (Table 2).

**Table 2.** Nonsurgical Procedures With Decreasing Interest Trends When Comparing Before and After the Start of the COVID-19 Pandemic.

Category	Statistic	*P*-value	Effect size	Effect magnitude
Chemical peel				
TCA skin peel^*^ + TCA peel^*^ + TCA chemical peel^*^	5097	.203	0.0666	Low
Filler				
Volbella^®^ + Voluma^®^ + Vollure^®^ + Juvederm^®^ + Ultra XC Juvederm^®^	3771	1	0.3094	Moderate
Bellafill^®^	2037	1	0.6269	High
Renuva^®^	4771	.943	0.1262	Low
Dermaplaning				
Microdermabrasion	1009	1	0.8153	High
Laser Lipo				
Smartlipo^®^	5430	.473	0.0056	Low
Sculpsure^®^	1724	1	0.6843	High
Coolsculpting^®^				
Coolsculpting^®^ + Coolsculpt^®^	2438	1	0.5536	High
Skin laser treatment				
Fraxel^®^ laser resurfacing	4843	.922	0.1131	Low
Intense pulsed light skin treatment + photofacial	3234	1	0.4077	Moderate
Profractional^™^ laser	4582	.978	0.1609	Low
Cellulite treatment				
Cellfina^®^	4063	.999	0.2560	Low

* TCA = trichloroacetic acid

## Discussion

In 2020, ASPS reported that 13.2 million non-surgical procedures were performed, which was a 16% decrease from 2019 due to the COVID-19 lockdown policies and allocation of healthcare resources.^9^ However, as elective procedures were again offered to patients and with 76% of plastic surgeons reporting increased demand compared to before the pandemic, nonsurgical plastic surgery procedure interest followed and dramatically rose in 2021, as shown in our study.^9^ While the full 2021 ASPS report is not currently released, studies have shown that Google Trends may be a powerful predictor of public demand for procedures. It is feasible that the data described in this study may be able to predict incoming trends and changes. Furthermore, Google Trends provides data for colloquial terms which are omitted from annual Society reports, such as “newtox,” “lip flip,” and “liquid rhinoplasty.”

While Google Trends can provide data beyond the scope of these reports, examining Google Trends data in conjunction with ASPS or The Aesthetic Society (TAS) reports is insightful. Both ASPS and TAS rank neurotoxin procedures as the most common nonsurgical procedure in every age group, followed by soft tissue fillers.^9,16^ While both of these procedures maintained a large nonsurgical procedure volume at baseline as shown in the ASPS and TAS data reports, interest significantly increased at the onset of the pandemic. This builds upon a prior study examining nonsurgical plastic surgery procedures, which reported that search interest and surgical volume for Botox were both expanding prior to COVID-19.^12^ As interest in Botox generally increased, the “lip flip” and Dysport brand of injections showed the steepest increases over other botulinum toxin brands. The “lip flip,” popular from social media, is an injection into the superior orbicularis oris intended to relax the upper lip, and embodies the rising popularity of above-the-shoulder procedures in response to the pandemic.^17^

Fillers had the largest magnitude increase in interest from pre- to post-COVID-19 lockdown. An increase was observed across different injection sites and filler brands, including Restylane and Versa. Searches in layman’s terms, which circulate more on social media or among the general public, such as tear trough filler, jawline filler, or lip filler, enjoyed large increases in interest. Searches consisting of anatomical or technical terms, such as marionette lines and nasolabial fold fillers, did not significantly vary during the pandemic. This accentuates the importance of using plastic surgery terms that the general public can understand. Specifically, the increased interest in tear trough fillers may be due to the increased desire for patients to appear less tired, as surveys reported increasing from 17% to 73%.^9,18^ Out of all the nonsurgical procedures, “lip flip” or lip Botox and lip filler had the highest effect size and statistically significant increases from pre to post-COVID-19 lockdown periods.

The procedures highest in volume trailing Botox and fillers differed between ASPS and TAS before and after the start of the COVID-19 pandemic. Comparing the 2021 ASPS, TAS, and AAFPRS data, nonsurgical skin tightening and facial skin care treatments, such as chemical peels and laser skin treatments, were consistent with our results, which showed an increased interest in Morpheus8, chemical peels, “diamond facials,” microdermabrasion, microneedling with radiofrequency, and microneedling with platelet-rich plasma.^9,16,18^

However, consistent with ASPS data but not with TAS data, our results showed an increase in interest of specific types of nonsurgical fat reduction and body contouring, namely laser liposuction and Emsculpt. Interestingly, there was no change in interest in CoolSculpting. In both datasets, these different types are grouped into the same category.

Additionally, searches for the thread lift were among the most steeply increasing, and boasted one of the largest effect sizes. However, it is not well-recorded in the ASPS or TAS datasets, and is either omitted or grouped under “Other.”

The observed trends may be attributed to shifting patient interests during or in reaction to the pandemic. A recent survey from ASPS showed that patients are more likely to do a procedure for themselves now, for increased confidence and to feel good, and that patients have more discretionary income after not paying for as much travel. COVID-19 safety guidelines formed an environment that was not conducive to invasive procedures, due to the time commitment or cost to patients. Conversely, nonsurgical procedures demanded a shorter recovery period, were easier to fit into a busy lifestyle, and were more budget friendly.^9^ The conveniences of nonsurgical procedures, combined with the influences of social media or frequent video conferencing, resulted in a heightened demand for these procedures, as reflected in the most recent AAFPRS and ASPS reports. A study showed that the amount of time on social media and viewing plastic surgery-related material profoundly impacted people’s decisions to undergo aesthetic procedures in the future.^19^ Additionally, for facial procedures, the lockdown afforded patients the opportunity to post-operatively recover at home while still being able to work, with surveys showing that 92% of patients finding this a main benefit.^9,18,20^ Patient have reported that increased video-calling during and after the pandemic may have encouraged increased facial surgeries directly.^9,18^ Above-the-shoulder procedures ballooned in interest after February 2020, which is reportedly related to video conferencing, called the Zoom effect.^17^ Surveys have confirmed that patients want to look better on screen on both Zoom, on Tik Tok, and in selfies, with 79% of facial plastic surgeons reporting that this was a main driver for patients in consultation.^18^

The limitations of our study include the innate limitations of the Google Trends Search Tool. Since Google Trends data are normalized to the highest search volume with the search input, time period, and geographic location, it cannot be compared to other data points. This allows only for the ability to examine the directionality and slope of trends and interest over time, not actual volume of searches in comparison. As an example, a 65 datapoint from Botox does not mean that Botox is more popular than a 55 datapoint from lip filler since the data do not convey information about volume, and inputs were normalized separately. Additionally, Google Trends does not capture all interest, including those who use different search websites, those who are interested but do not search on Google, those who use social media as a way to look up procedures instead of Google, and those who do not have access to technology. Furthermore, while our study tried to broadly capture as many colloquial and clinical surgical procedure terms as possible, there may be some surgical procedures that are described and searched in terms that were not captured in this study.

However, an understated benefit of Google Trends is the specificity of data that can be obtained. The exact site of injection, type of procedure, brand, or specific technology can be queried into the Search Tool. In this study, distinctions between brands of Botox, procedures described in layman’s terms, types of filler, and methods of nonsurgical fat reduction illuminated trends that were not captured by ASPS or The Aesthetic Society reports.

## Conclusions

Consistent with other studies evaluating plastic surgery trends after COVID-19, interest in the majority of nonsurgical procedures increased after the COVID-19 lockdown, with facial procedures showing the most increase in interest, with the steepest trends from neurotoxins, fillers, thread lift, and skin treatments. While Botox and fillers have always been the most common nonsurgical procedures, these 2 nonsurgical procedures showed an even greater increase in interest after the start of the COVID-19 pandemic. Additionally, interest in lip enhancement and skin-improving procedures has dramatically increased. Plastic surgeons can use the Google Trends tool to aid in predicting the next trend and anticipating increases in specific procedure, such as lip Botox, or to invest in new technology, such as Morpheus8. Our study has shown that people’s interests and preferences around nonsurgical plastic surgery procedures have changed after the start of the pandemic.
